# Sleep Health Characteristics among Adults Who Attempted Weight Loss in the Past Year: NHANES 2017–2018

**DOI:** 10.3390/ijerph181910170

**Published:** 2021-09-28

**Authors:** Marquis S. Hawkins, Michele D. Levine, Daniel J. Buysse, Kaleab Z. Abebe, Wei-Hsin Hsiao, Kathleen M. McTigue, Esa M. Davis

**Affiliations:** 1 Department of Epidemiology, University of Pittsburgh, Pittsburgh, PA 15261, USA; weh57@pitt.edu (W.-H.H.); kmm34@pitt.edu (K.M.M.); 2 Department of Psychiatry, University of Pittsburgh, Pittsburgh, PA 15261, USA; levinem@upmc.edu (M.D.L.); buyssedj@upmc.edu (D.J.B.); 3 Department of Medicine, University of Pittsburgh, Pittsburgh, PA 15261, USA; kza3@pitt.edu (K.Z.A.); emd55@pitt.edu (E.M.D.)

**Keywords:** sleep health, obesity, weight loss, surveillance

## Abstract

Background: The purpose of this study was to characterize sleep health in adults who attempted weight loss in the prior year. Methods: We analyzed data from the National Health and Nutrition Examination Survey 2017–2018 exam cycle. We included 4837 US adults who did (*n* = 1919) or did not (*n* = 2918) attempt weight loss in the past year. Participants self-reported their sleep regularity, satisfaction, sleepiness, timing, and duration, which we defined as “good” based on the prior literature. We characterized sleep health by weight loss attempts status, current BMI and weight change among participants who attempted weight loss. Results: On average, participants reported good sleep health in 3.21 ± 1.14 out of the five sleep domains. A total of 13% of participants had good sleep health in all five domains. The prevalence of sleep regularity (52%) was lowest, and the prevalence of infrequent sleepiness was highest (72%), relative to other sleep domains. In models adjusting for BMI, sleep health was similar in participants who did and did not attempt weight loss. Among adults who attempted weight loss, good sleep health was inversely associated with current BMI and self-reported weight change. Discussion: This study’s findings highlight the importance of considering sleep health when engaging with adults attempting weight loss.

## 1. Introduction

The US Preventive Service Task Force (USPSF) recommends that clinicians offer or refer adults with obesity to a lifestyle intervention that addresses diet and physical activity [1]. Consistent with the USPSF’s recommendations, most adults who attempt weight loss choose to modify their diet and physical activity levels [2]. For example, Han et al. characterized trends in weight loss strategies used by a nationally representative sample of US adults from 1999 to 2016 [2]. The authors found that between 24 to 42% of US adults attempted weight loss in the past year. The most common weight loss strategies were reduced food consumption, increased exercise, and increased water intake [2]. The authors noted that monitoring diet and exercise behaviors in adults attempting weight loss are essential, because these behaviors are associated with weight loss success. However, sleep health is independently associated with obesity [3] and weight loss success [4], but is rarely characterized among adults attempting weight loss, particularly in nationally representative samples.

Some researchers have suggested that sleep health should be considered an essential component of screening and counseling for weight management for several reasons [5]. First, sleep health characteristics including sleep duration, sleep timing, sleepiness and sleep regularity, are associated with weight gain and obesity independent of physical activity and diet [3,6]. Second, poor sleep health can influence physical activity and dietary intake, common weight loss intervention targets. For example, sleep disturbances are associated with reduced physical fitness [7], lower exercise frequency [8], and physical inactivity [9,10,11,12]. Short sleep duration also affects brain reward systems [13,14], leading to increased hunger, increased cravings for energy-dense foods (e.g., high fat, high sugar content), larger portion sizes, and increased energy intake [15]. Lastly, there is evidence from clinical trial data that poor sleep health can reduce the effectiveness of diet and physical activity weight management interventions [4,16,17,18,19,20,21,22,23,24]. For example, in the LIFE study (83% women), participants who slept >6 h/night were twice as likely to have successful weight loss than those who slept <6 h/night [4].

Given the relationship between sleep, diet, physical activity and weight loss, it is important to monitor sleep health among adults attempting weight loss. Such research may help identify sleep health patterns that may be conducive to weight loss success, and others that may make weight loss more difficult and perhaps merit intervention to promote the success of diet and exercise strategies. The National Health and Nutrition Examination Survey (NHANES) collects data on weight loss attempt history and sleep health, which provides an opportunity to fill this research gap. The purpose of this study was to characterize sleep health in a nationally representative sample of adults who reported attempting weight loss in the prior year. We also characterized sleep health indicators by current BMI, self-reported difference in prior-year weight and current weight (i.e., weight change).

## 2. Materials and Methods

NHANES is a cross-sectional study of non-institutionalized US residents conducted by the National Center for Health Statistics of the CDC. NHANES uses a stratified, multistage probability design to obtain a nationally representative sample of the US population. Non-Hispanic Black and Hispanic individuals, persons 60 years of age and older, and low-income individuals are over-sampled to produce nationally representative estimates [25]. Trained interviewers conducted health interviews in the participant’s home to collect data on demographics, weight history, and sleep habits. Anthropometric data (i.e., weight, height, waist circumference) were collected in a mobile examination center [26]. The National Center for Health Statistics institutional review board approved the NHANES protocol. All participants provided written informed consent at the time of the household interview. We used data from the 2017–2018 exam cycle, the most recently available NHANES exam cycle, for this analysis. We included 4837 US adults who did (*n* = 1919) or did not (*n* = 2918) attempt weight loss in the past year.

### 2.1. Sleep Health Indicators

We selected sleep health indicators that were consistent with the sleep health framework proposed by Buysse [27]. Sleep satisfaction was determined using doctor-reported sleep problems or trouble. Although doctor-reported sleep problems is not a direct measure of sleep satisfaction, prior research has shown that reporting sleep problems to a doctor is associated with lower sleep satisfaction [28]. Good sleep health in this domain was defined as not reporting sleep problems or trouble to a doctor. Sleepiness was assessed by asking participants, “How often [do you] feel overly sleepy during the day?” Participants’ responses ranged from never, rarely (once a month), sometimes (2 to 4 times/month), often (5 to 15 times/month), and almost always (16 to 30 times/month). Good sleep health in this domain was defined as never, rarely or sometimes feeling overly sleepy. For simplicity, we will refer to good sleep health in this domain as infrequent sleepiness. Sleep timing was based on sleep midpoint, the halfway point between the participants’ bedtime and wake time. Participants reported their weekday/workday and weekend/non-workday bedtimes and wake times separately. We defined sleep timing as a weighted average of weekday/workday and weekend/non-workday sleep midpoint. Good sleep health in this domain was defined as a sleep midpoint between 2:00 a.m., and 4:00 a.m. Early and late sleep timing was defined as a sleep midpoint before or after 2:00 a.m. and 4:00 a.m., respectively. Sleep regularity was defined as the difference in sleep timing between the weekday/workday and weekend/non-workdays. Good sleep health in this domain was defined as a less than 60-min difference in weekday/workday and weekend/non-workday sleep midpoint. Sleep duration was estimated by taking the difference between self-reported usual bedtime and waketime. Participants reported their weekday/workday and weekend/non-workday bedtime and wake times separately. We computed sleep duration as a weighted average of weekday and weekend sleep duration. Consistent with the American Academy of Sleep Medicine guidelines, we defined good sleep health in this domain as reporting from 7 to 9 h of sleep per night [29]. Finally, we created an overall sleep health composite score as the sum of good sleep health indicators in each domain. The scores ranged from 0 to 5, with higher scores indicating better sleep health.

### 2.2. Weight History in the Prior Year and Current BMI

To determine if participants attempted weight loss in the past year, they were asked, “During the past 12 months, have you tried to lose weight?” We categorized participants into groups based on weight loss attempt status (yes vs. no). Additionally, we created weight change groups based on participants’ self-reported current weight and weight one year prior (i.e., weight loss, weight maintenance, weight gain). Current body mass index (BMI) was calculated as weight in kilograms (kg) divided by height in meters squared (m^2^). Trained technicians measured the participant’s weight using a digital scale, with the participant dressed in light clothing. Participants’ height was measured while standing, using a stadiometer. Normal weight, overweight, and obesity was defined according to the WHO criteria [30].

### 2.3. Descriptive Characteristics

Demographic characteristics were collected through self-report during the household examination. They included age, race/ethnicity (non-Hispanic White, non-Hispanic Black, Mexican American, Other Hispanic), number of children in the household (0, 1, 2, 3+), age of the youngest child (continuous), marital status (married/living with a partner, single), education (<high school, high school or GED, some college and beyond), employment status (not working, part-time (<35 h/week), and full-time (≥35 h/week)) and family monthly income. Family income was categorized using the poverty index ratio (PIR) created by the Department of Health and Human Services poverty guidelines, which take family size into account [31]. Family income categories were as follows: PIR < 1.3 = low income; PIR 1.3 to 3.5 = middle income; PIR > 3.5 = high income.

Physical activity was assessed using a modified Global Physical Activity Questionnaire [32]. Interviewers asked participants if they engaged in any vigorous or moderate-intensity sports, fitness, or recreational activity in a typical week. Participants who answered “yes” were further queried on the typical frequency and duration of these activities. We estimated the total duration of moderate- and vigorous-intensity physical activity by taking the product of the reported frequency and duration of activities. Consistent with national guidelines, we defined meeting guidelines for physical activity as reporting at least 150 min of moderate–intensity physical activity, 75 min of vigorous-intensity physical activity, or an equivalent combination of both [33]. Two 24 h food recalls were used to assess dietary intake. We calculated the Healthy Eating Index scores to provide an estimate of overall dietary quality [34]. Participants also reported their total intake of plain water.

### 2.4. Statistical Analysis

Descriptive statistics including means, standard deviations (SD), frequencies and proportions were calculated for demographic characteristics. Student’s *t*-tests and Chi-square tests were used to compare demographic characteristics by reported weight-loss attempts (yes vs. no) in the past year.

For our primary analysis, we reported means (SD) or medians (interquartile range) for the continuous sleep health dimensions. We then used Poisson regression with a robust variance to estimate the prevalence ratios (PR) and 95% confidence intervals (CIs) of each sleep health indicator by reported weight loss attempts. Estimating PR with Poisson regression is preferrable to reporting odds ratios from logistic regression models when the outcomes are frequent [35]. We used linear regression to compare participants’ sleep health composite scores by reported weight loss attempts. For each analysis, the reference group was participants who did not attempt weight loss. Model 1 adjusted for demographic characteristics including age, gender, race/ethnicity, education, income, and marital status. Model 2 additionally adjusted for current BMI.

For our secondary analysis, we examined associations between current measured BMI (i.e., normal weight, overweight, obesity class I, obesity class II) and self-reported weight change (i.e., weight loss, weight maintenance, weight gain), with sleep health a among participants who reported attempting weight loss. We used a similar analytic approach to that described for the primary analysis. For the analysis of weight change and sleep health, the reference group was participants who reported weight loss.

All analyses were conducted in the R statistical environment. We used the “survey” package to account for the complex multistage probability sampling design for all analyses. We report weighted sample size and prevalence estimates. The samples sizes reported in the tables reflect the number of people in the US population that our data represent. All analyses were considered statistically significant at an alpha of 0.05 and no adjustments were made for multiplicity.

## 3. Results

Approximately 43% of adults reported attempting weight loss in the past year. A higher percentage of participants who attempted weight loss (vs. did not) were women, were married, had greater than a high school education, had a higher income, and had overweight or obesity. Adults who attempted weight loss were also more physically active, had slightly higher dietary quality, and reported higher water intake than adults who did not attempt weight loss (Table 1).

In the overall sample, only 13% of participants had good sleep health in all five assessed sleep domains. Participants, on average, had good sleep health in 3.21 ± 1.14 of the five sleep domains. The prevalence of good sleep health in the overall sample varied by sleep domain, ranging from 52% to 72%. Specifically, the median difference in weekday/workday and weekend/non-workday sleep midpoint (i.e., sleep regularity) was 45 min (IQR = 0, 90 min), with 54% reporting good sleep regularity (<60 min) (Table 2). In contrast, 72% of participants reported infrequent sleepiness. In unadjusted models, adults who attempted weight loss (vs. did not) reported lower sleep satisfaction and more frequent sleepiness, but had a higher prevalence of good sleep timing (Table 2).

In models adjusting for age, race, gender, education, income, and marital status, there were no statistically significant differences in sleep health characteristics between participants who did and did not attempt weight loss except for sleep satisfaction (Table 3). Participants who attempted weight loss (vs. did not) had a 10% (PR = 0.89, 95% CI = 0.84 to 0.96) lower prevalence of sleep satisfaction than participants who did not attempt weight loss. However, differences in sleep satisfaction between the groups were attenuated and no longer statistically significant after additionally adjusting for current BMI (Table 3).

Among participants who attempted weight loss in the past year, sleep health characteristics varied by current BMI (Table 4). In unadjusted models, participants with obesity (vs. normal BMI) had a lower prevalence of good sleep satisfaction, infrequent sleepiness, good sleep timing, and had lower overall sleep health (Table 4). Participants with obesity (vs. normal BMI) also had a lower prevalence of good sleep regularity and good sleep duration, but these differences were not statistically significant.

Differences in sleep health between BMI groups remained after adjustment for demographic characteristics (Table 5). Specifically, each increase in current BMI category was associated with an approximately 10% lower prevalence of sleep satisfaction (PR = 0.90, 95% CI = 0.84 to 0.96) and infrequent sleepiness (PR = 0.92, 95% CI = 0.88 to 0.98), and a lower overall sleep health score.

Lastly, we examined the prevalence of good sleep health among participants who reported weight loss by weight change (Table 6). In the unadjusted models, the prevalence of good sleep health was similar across weight change groups, except for good sleep timing and overall sleep health (Table 6). Participants who lost weight had a higher prevalence of good sleep timing and a slightly higher overall sleep health score.

In models adjusting for demographic characteristics, participants who lost weight had better sleep health in multiple domains (Table 7). Specifically, participants who maintained their weight in the prior year had a lower prevalence of good sleep regularity (PR = 0.88, 95% CI = 0.80 to 0.97) and good sleep timing (PR = 0.83, 95% CI = 0.74 to 0.93) than participants who lost weight. Additionally, participants who gained weight in the past year had a lower prevalence of sleep satisfaction (PR = 0.89, 95% CI = 0.82 to 0.97) than participants who lost weight (Table 7).

## 4. Discussion

Sleep health is independently associated with obesity and weight gain but is often overlooked in studies that characterize weight loss strategies in adults. Therefore, we characterized sleep health in a nationally representative sample of US adults who did and did not attempt to lose weight in the prior year. In the overall sample, we found that nearly half did not meet thresholds for good sleep regularity and nearly 40% reported early or late sleep timing. Consistent with prior research, a substantial proportion of the population did not meet recommendations for sleep duration. Sleep satisfaction was less common in adults who attempted weight loss in the past year compared to adults who did not, which was explained by differences in current BMI. Among adults who attempted weight loss, the prevalence of good sleep health was inversely associated with current BMI and weight change. Participants reporting weight loss had better sleep regularity, timing, and satisfaction than participants who maintained or gained weight. These findings highlight the burden of poor sleep health in a variety of domains in a US adult population and particularly among those attempting weight loss.

This study’s results are novel because they are the first to characterize sleep health across multiple domains in a nationally representative sample of US adults. The majority of prior studies to characterize sleep in nationally representative sampled focused on sleep duration, documenting the high prevalence of sleep insufficiency. A few studies have reported the average sleep timing in adults using data from NHANES [36], but did not characterize the heterogeneity of sleep timing across the groups used in this study. Additionally, to the authors’ knowledge, this is the first study to characterize the prevalence of good sleep health across several domains in a nationally representative sample of adults who attempted weight loss. This study’s findings have important implications for designing effective weight loss strategies. Specifically, it is important to understand the magnitude of the problem you intend to solve in early stages of intervention development. While insufficient sleep duration is well documented among nationally representative samples of adults, sleep health in other domains, and specifically among people who attempted weight loss, is less understood. We found that a substantial proportion of adults attempting weight loss keep irregular sleeping schedules. Since prior research provides evidence that sleep regularity is associated with obesity and weight loss maintenance [37,38], weight loss interventions should be aware of the potential for poor sleep regularity in their population and should measure, and potentially address, irregular sleep patterns.

There are several limitations worth considering when interpreting this study’s results. As previously mentioned, we only have measures of the participants’ current sleep, which may not reflect their sleep prior to or during their weight loss attempt. Relatedly, our study is limited by its cross-sectional design. However, despite not being able to identify the temporal relationship between sleep health and weight loss attempts, we were able to document the burden of poor sleep health across several domains and generate hypotheses that can be further evaluated in a future prospective study. Future research using prospective designs will provide additional information on sleep patterns before and during weight loss attempts and how they impact weight loss success. Another limitation is that there is no consensus on indicators of good sleep health for some sleep domains. Specifically, we do not have expert consensus for indicators of good sleep timing and regularity. Changing thresholds for sleep health indicators would vary the prevalence of good sleep health across domains and possibly their association with BMI and weight change. We used thresholds deemed reasonable by our team of experts and from prior studies [27,39,40]. Thus, we believe our thresholds are reasonable based on the current state of knowledge. Additionally, the study was limited by its reliance on self-report to measure all indicators of sleep health. Self-report is useful for capturing some sleep health indicators, such as sleepiness and satisfaction. However, self-report differs from direct measures for estimating sleep health in other domains, such as sleep duration [41]. Despite the limitations of self-reporting, and its potential for over- or underestimating sleep in some domains, much of the literature supporting the American Academy of Sleep Medicine, Sleep Research Societies, and the National Sleep Foundation’s sleep health recommendations are based on self-reported sleep data [29,42,43]. Additionally, self-reports are more feasible than direct measures of sleep in large population-based studies, allowing us to derive more representative population estimates. Lastly, participants’ perceptions of their sleep are associated with health outcomes independent of their objectively measured sleep [44]. Self-report and objective measures of sleep each have value for estimating various dimensions of sleep health and future studies should incorporate both assessment tools. Despite the limitations, the strength of this study is that it is a large, diverse sample of US adults with data on sleep across several domains.

## 5. Conclusions

In conclusion, the prevalence of good sleep health, across multiple domains, was low in a substantial proportion of US adults. Among adults who attempted weight loss in the past year, sleep health was particularly poor among those who had higher BMI’s or did not lose weight in the past year. This study’s findings highlight the importance of considering sleep health when engaging with adults attempting weight loss.

## Figures and Tables

**Table 1:** Descriptive characteristics by weight loss attempt status.

Variable	Overall *N* = 206,714,810 ^1^	Attempted Weight Loss		*p*-Value ^2^
		No *N* = 117,391,936 ^1^	Yes *N* = 89,322,874 ^1^	
Age, years	48.5 (17.5)	49.0 (18.2)	47.8 (16.4)	0.34
Gender				<0.001
Men	49%	54%	43%	
Women	51%	46%	57%	
Race/Ethnicity				0.12
Non-Hispanic White	63%	64%	61%	
Non-Hispanic Black	11%	12%	11%	
Hispanic	16%	14%	17%	
Other	10%	10.0%	11%	
Married	63%	60%	66%	0.031
Education				0.005
<High school	10%	11%	8.5%	
High school	19%	21%	17%	
>High school	71%	68%	75%	
Working Status				0.29
Not working	40%	41%	38%	
Part-time	14%	15%	14%	
Full-time	46%	44%	48%	
Income Level				<0.001
Low	20%	24%	16%	
Middle	36%	38%	34%	
High	44%	39%	50%	
General Health Status				0.19
Poor or fair	41%	42%	39%	
Good	57%	55%	59%	
Very good or excellent	2.4%	2.4%	2.4%	
BMI, kg/m^2^	29.4 (7.3)	27.5 (6.5)	32.0 (7.4)	<0.001
BMI				<0.001
Normal weight	29%	41%	13%	
Overweight	31%	30%	33%	
Class I Obesity	21%	17%	26%	
Class II Obesity	19%	13%	28%	
Depressive Symptoms	1.0 (0.0, 4.0)	1.0 (0.0, 4.0)	2.0 (0.0, 4.0)	0.006
MVPA, min/week	40.0 (0.0, 210.0)	0.0 (0.0, 210.0)	80.0 (0.0, 225.0)	<0.001
Met PA Guidelines	36%	33%	40%	0.002
Healthy Eating Index	51.3 (42.0, 61.5)	50.0 (41.0, 60.3)	52.8 (43.1, 63.0)	0.006
Water Intake, gm	997.9 (360.0, 1814.9)	887.0 (279.7, 1740.0)	1020.0 (507.0, 1920.0)	0.004

MVPA = Moderate-Vigorous Intensity Physical Activity; PA = Physical Activity; BMI = Body Mass Index; ^1^ Median (IQR) or %; ^2^ Wilcoxon rank-sum test for complex survey samples; chi-squared test with Rao and Scott’s second-order correction.

**Table 2:** Sleep health characteristics by weight loss attempt status.

Variable	Overall *N* = 206,714,810 ^1^	Attempted Weight Loss		*p*-Value ^2^
		No *N* = 117,391,936 ^1^	Yes *N* = 89,322,874 ^1^	
Sleep Regularity, min	45 (0, 90)	30 (0, 90)	45 (0, 90)	0.33
Good Sleep Regularity ^3^	54%	55%	53%	0.48
Sleep Satisfaction ^3^	70%	73%	67%	0.003
Infrequent Sleepiness ^3^	72%	74%	70%	0.041
Sleep Midpoint, a.m.	2:55 (2:06, 3:45)	2:55 (2:02, 3:47)	2:55 (2:10, 3:45)	0.81
Good Sleep Timing ^3^	59%	58%	62%	0.045
Early Sleep Timing, <2 a.m.	21%	23%	19%	0.056
Late Sleep Timing, >4 a.m.	20%	20%	20%	0.60
Good Sleep Duration ^3^	65%	63%	66%	0.25
Overall Sleep Health ^4^	3.21 (1.14)	3.24 (1.15)	3.18 (1.14)	0.27

^1^ Median (IQR), %, or Mean (SD); ^2^ Wilcoxon rank-sum test for complex survey samples; chi-squared test with Rao and Scott’s second-order correction; ^3^ Indicators (%yes) of good sleep health in the respective dimension; ^4^ Number of good sleep health indicators met.

**Table 3:** Prevalence ratios for good sleep health indicators by weight loss attempt status.

Characteristic	Model 1			Model 2		
	PR ^1^	95% CI ^1^	*p*-Value	PR ^1^	95% CI ^1^	*p*-Value
Sleep Regularity						
Attempted Weight Loss, Yes vs. No	1.02	0.92, 1.13	0.7	1.03	0.93, 1.15	0.6
Sleep Satisfaction						
Attempted Weight Loss, Yes vs. No	0.90	0.84, 0.96	0.038	0.95	0.88, 1.01	0.2
Sleepiness						
Attempted Weight Loss, Yes vs. No	0.96	0.90, 1.02	0.2	1.02	0.96, 1.08	0.6
Sleep Timing						
Attempted Weight Loss, Yes vs. No	1.03	0.97, 1.08	0.4	1.03	0.97, 1.09	0.4
Sleep Duration						
Attempted Weight Loss, Yes vs. No	1.02	0.95, 1.09	0.7	1.02	0.95, 1.10	0.6

Model 1 adjusts for age, gender, race/ethnicity, education, income, marital status. Model 2 adjusted for Model 1 + BMI. ^1^ PR = Prevalence Ratio, CI = Confidence Interval.

**Table 4:** Sleep characteristics in participants who attempted weight loss by BMI status.

Variable	BMI Group				*p*-Value ^2^
	Normal Weight, *N* = 11,449,674 ^1^	Overweight, *N* = 29,322,973 ^1^	Obesity I, *N* = 23,088,355 ^1^	Obesity II, *N* = 24,512,433 ^1^	
Sleep Regularity, min	45 (0, 90)	45 (0, 75)	30 (0, 75)	60 (0, 105)	0.27
Good Sleep Regularity	56%	52%	58%	48%	0.35
Sleep Satisfaction	73%	73%	64%	58%	0.003
Infrequent Sleepiness	71%	80%	67%	62%	0.003
Sleep Midpoint, am	2:55 (2:26, 3:35)	2:47 (2:02, 3:30)	3:00 (2:08, 3:53)	2:58 (2:17, 4:00)	0.16
Good Sleep Timing	74%	64%	57%	58%	0.056
Early Sleep Timing, <2 am	12%	20%	21%	20%	0.31
Late Sleep Timing, >4 am	15%	16%	23%	23%	0.10
Good Sleep Duration	67%	67%	68%	62%	0.37
Overall Sleep Health	3.40 (1.08)	3.36 (1.09)	3.15 (1.12)	2.88 (1.18)	0.006

^1^ Median (IQR); %; Mean (SD); ^2^ Wilcoxon rank-sum test for complex survey samples; chi-squared test with Rao and Scott’s second-order correction.

**Table 5:** Prevalence ratios for good sleep health indicators among adults who attempted weight loss by BMI group.

Variable	PR ^1^	95% CI ^1^	*p*-Value	*p*-Value ^2^
Good Sleep Regularity				0.14
BMI Group				
Normal	—	—		
Overweight	0.85	0.64, 1.12	0.31	
Obesity I	0.91	0.74, 1.11	0.39	
Obesity II	0.79	0.62, 1.01	0.13	
Sleep Satisfaction				0.03
BMI Group				
Normal	—	—		
Overweight	0.99	0.84, 1.17	0.92	
Obesity I	0.87	0.72, 1.05	0.22	
Obesity II	0.76	0.60, 0.96	0.086	
Infrequent Sleepiness				0.03
BMI Group				
Normal	—	—		
Overweight	1.08	0.88, 1.32	0.49	
Obesity I	0.92	0.76, 1.10	0.41	
Obesity II	0.86	0.69, 1.06	0.22	
Good Sleep Timing				0.26
BMI Group				
Normal	—	—		
Overweight	0.89	0.76, 1.05	0.23	
Obesity I	0.79	0.64, 0.97	0.091	
Obesity II	0.86	0.69, 1.08	0.26	
Good Sleep Duration				0.83
BMI Group				
Normal	—	—		
Overweight	1.04	0.91, 1.18	0.59	
Obesity I	1.08	0.90, 1.30	0.46	
Obesity II	1.02	0.86, 1.21	0.80	

Adjusted for age, gender, race/ethnicity, education, income, marital status; ^1^ PR = Incidence Rate Ratio, CI = Confidence Interval; ^2^ *p*-value is for a test of trend.

**Table 6:** Sleep characteristics in participants who attempted weight loss by self-reported weight change.

Variable	Weight Change Group			*p*-Value ^2^
	Weight Loss, *N* = 18,298,545 ^1^	Weight Maintenance, *N* = 29,146,345 ^1^	Weight Gain, *N* = 41,877,984 ^1^	
Sleep Regularity, min	30 (0, 90)	45 (0, 90)	45 (15, 90)	0.24
Good Sleep Regularity	57%	53%	51%	0.37
Sleep Satisfaction	70%	65%	66%	0.50
Infrequent Sleepiness	70%	73%	68%	0.36
Sleep Midpoint, am	2:49 (2:15, 3:30)	2:55 (2:06, 3:51)	2:55 (2:09, 3:49)	0.32
Good Sleep Timing	70%	58%	60%	0.060
Early Sleep Timing, <2 am	15%	20%	20%	0.28
Late Sleep Timing, >4 am	15%	22%	20%	0.24
Good Sleep Duration	68%	71%	62%	0.10
Overall Sleep Health	3.36 (1.19)	3.22 (1.11)	3.07 (1.12)	0.071

^1^ Median (IQR); %; Mean (SD); ^2^ Wilcoxon rank-sum test for complex survey samples; chi-squared test with Rao and Scott’s second-order correction.

**Table 7:** Prevalence ratios for good sleep health indicators among adults who attempted weight loss by self-reported weight change in the past year.

Variable	PR ^1^	95% CI ^1^	*p*-Value
Good Sleep Regularity			
Weight Change			
Weight Loss	—	—	
Weight Maintenance	0.88	0.80, 0.97	0.048
Weight Gain	0.98	0.87, 1.10	0.76
Sleep Satisfaction			
Weight Change			
Weight Loss	—	—	
Weight Maintenance	0.95	0.81, 1.12	0.56
Weight Gain	0.89	0.82, 0.97	0.043
Infrequent Sleepiness			
Weight Change			
Weight Loss	—	—	
Weight Maintenance	1.08	0.93, 1.26	0.34
Weight Gain	1.03	0.89, 1.18	0.73
Good Sleep Timing			
Weight Change			
Weight Loss	—	—	
Weight Maintenance	0.84	0.74, 0.94	0.031
Weight Gain	0.90	0.79, 1.02	0.17
Good Sleep Duration			
Weight Change			
Weight Loss	—	—	
Weight Maintenance	1.01	0.87, 1.17	0.89
Weight Gain	0.88	0.78, 0.99	0.090

Adjusted for age, gender, race/ethnicity, education, income, marital status; ^1^ PR = Prevalence Ratio, CI = Confidence Interval.

## Data Availability

The data analysis used publicly available data which can be accessed at: https://wwwn.cdc.gov/nchs/nhanes/continuousnhanes/default.aspx?BeginYear=2017 (accessed on 1 May 2021).
